# FeV LDH Coated on Sandpaper as an Electrode Material for High-Performance Flexible Energy Storage Devices

**DOI:** 10.3390/polym15051136

**Published:** 2023-02-24

**Authors:** Jihyeon Park, Youngsu Kim, Narasimharao Kitchamsetti, Seungju Jo, Seungjae Lee, Jubin Song, Wook Park, Daewon Kim

**Affiliations:** 1 Department of Electronics and Information Convergence Engineering, Kyung Hee University, Yongin 17104, Republic of Korea; 2 Department of Electronic Engineering, Kyung Hee University, Yongin 17104, Republic of Korea; 3 Institute for Wearable Convergence Electronics, Kyung Hee University, Yongin 17104, Republic of Korea

**Keywords:** sandpaper, layered double hydroxide, hierarchical structure, hybrid supercapacitor, flexible supercapacitor

## Abstract

Recently, considerable research efforts to achieve advanced design of promising electroactive materials as well as unique structures in supercapacitor electrodes have been explored for high-performance energy storage systems. We suggest the development of novel electroactive materials with an enlarged surface area for sandpaper materials. Based on the inherent micro-structured morphologies of the sandpaper substrate, nano-structured Fe-V electroactive material can be coated on it by facile electrochemical deposition technique. A hierarchically designed electroactive surface is covered with FeV-layered double hydroxide (LDH) nano-flakes on Ni-sputtered sandpaper as a unique structural and compositional material. The successful growth of FeV-LDH is clearly revealed by surface analysis techniques. Further, electrochemical studies of the suggested electrodes are carried out to optimize the Fe-V composition as well as the grit number of the sandpaper substrate. Herein, optimized Fe_0.75_V_0.25_ LDHs coated on #15000 grit Ni-sputtered sandpaper are developed as advanced battery-type electrodes. Finally, along with the negative electrode of activated carbon and the FeV-LDH electrode, it is utilized for hybrid supercapacitor (HSC) assembly. The fabricated flexible HSC device indicates high energy and power density by showing excellent rate capability. This study is a remarkable approach to improving the electrochemical performance of energy storage devices using facile synthesis.

## 1. Introduction

In recent years, supercapacitors (SCs) have attracted widespread research attention as energy storage technologies. Due to their numerous advantages, such as long cycle life, high power density, and rapid charge-discharge capability, advanced energy storage and conversion systems using SC devices are being actively studied as substitutes for rechargeable batteries [1,2]. Specifically, designs of battery-type electrode materials for high power and energy density have been used to attain the apparent merits of both supercapacitors and rechargeable batteries. Thus, the development of advanced electroactive materials is the critical issue in achieving high performance energy storage systems [3,4].

Among the various key parameters that can be used to enhance electrochemical performance, considerable research efforts have been devoted to finding new and advanced materials and unique structures for SC electrodes [5,6,7,8,9]. First, improvements in ion/electron diffusion have enhanced the electrochemical performance. It is well known that the unique structural feature of enhanced surface area of electroactive materials is the key to achieving superior electrochemical performance [10,11,12]. According to the energy storage mechanism of battery-type supercapacitors, electrochemical ion/charges can be faradaically stored through fast and reversible redox reactions at the interface of electroactive materials and electrolyte. According to this mechanism, the enlarged surface area of electroactive materials is the crucial factor to facilitate the charge storage in battery-type electrodes [13,14]. In hybrid supercapacitors (HSCs), battery-type materials such as NiO and Co_3_O_4_ [15,16,17,18] exhibit higher electrochemical performance than those of pseudocapacitive materials (i.e., RuO_2_) [19,20]; this higher capacity may provide abundant activation sites and thereby enhance the charge storage performance. In order to achieve the goal of high-performance, we studied numerous hybrid or composite materials, including pseudo-materials such as conducting polymers. Conducting polymers include polyaniline, poly [3,4-ethylenedioxythiophene], polypyrrole, and polythiophene, which have been highly studied because of their low cost, environmental stability, and high electrical conductivity. In comparison with other electrode materials, conductive polymers attracted much attention due to their good flexibility, easy processing, and the polymer film thickness and composition could be attained with good control [9]. Furthermore, with the insertion and release of charged ions in the charging and discharging conditions, the expansion and contraction of conducting polymers will be under the action of charges and ions. This process often leads to the degradation of the cycle stability performance of the electrode material [1,2]. Taking the above problems into consideration, the design of proper electrode materials has been regarded as a feasible solution.

Moreover, numerous studies have been conducted on earth-abundant elements such as transition metal nitrides [21,22], sulfides [23,24], oxides [25], and hydroxides [26]. Among them, earth-abundant iron hydroxides are promising electroactive materials for SC electrodes. The characteristics of low cost, natural abundance, environmental benignity, and high activity in alkaline media favor its wide application. However, the poor conductivity (10^−5^ S/cm) of iron hydroxide hinders its practical application to supercapacitors [27]. Thus, various research efforts have been suggested to improve the conductivity, including tuning of the electronic structure, morphological optimization, composition, and interface engineering, all of which have been reported to increase the activation sites for charge storage [28,29]. Due to their multiple valence states and the various structures of their compounds, vanadium oxides have been extensively studied as favorable alternatives to HSCs. Even better than single-component vanadium oxides, iron vanadate hydroxide, which has richer redox chemical kinetics, can be utilized with multiple metal ions [30,31]. Iron vanadate hydroxide as an electrode for HSCs shows interfacial effects and enhanced ion/electronic conductivity, which are known to improve the electrochemical activity.

The rational design of electrode materials via a simple and favorable approach is vital for the fabrication of highly efficient electrochemical energy storage devices. In this regard, the nanoscale size of iron vanadate hydroxide allows the formation of larger exposed active sites, shortening the ion diffusion path compared with that of its bulk counterparts. In the past, numerous studies have reported that nano-size vanadates achieved better electrochemical performance than bulk electrodes in HSCs [32,33]. Various preparation methods of nanoscale-sized materials, including hydrothermal, electrospinning, and wet chemical methods, have been developed for the synthesis of vanadates with diverse morphologies. For instance, Ni_3_V_2_O_8_@Co_3_V_2_O_8_ composite [34], Ni_3_V_2_O_8_/GO composite [35], 3D Co_3_V_2_O_8_ with porous rose-like structure [36], and Ni_3_V_2_O_8_/NiO nanocomposite [37] have been prepared for use as electrode materials to obtain superior electrochemical performance in the field of SCs.

A rational design and facile synthetic process for vanadate-based binary metal oxides/hydroxides are thus favorable approaches to enhancing supercapacitor performance. Herein, the electrochemical performance of unique hierarchical FeV hydroxide structures decorated over nano/micro-sandpaper substrates is reported for the first time. The entire synthesis was carried out using a simple and viable electrochemical deposition (ECD) technique. As a result, nano-flakes/nano-spheres of Fe-V layered double hydroxides (LDHs) were hierarchically grown on Ni-sputtered sandpaper substrate. By tuning the unique surface morphology, these novel nanostructures on conductive sandpaper supported large amounts of electrolyte ion diffusion, resulting in enhanced electrochemical performance and high-rate capability. For the efficient decoration of Fe-V on the sandpaper substrate, optimization of Fe-V nanostructures as well as the determination of the proper grit-number of the sandpaper substrate were successfully carried out. It was found that Fe0_.75_V_0.25_ LDHs grown on #15000 grit sandpaper exhibited superior electrochemical performance. Furthermore, the assembled HSC device delivered a higher energy and power density of 0.123 mWh/cm^2^ and 23.8 mW/cm^2^ at the current densities of 2 and 18 mA/cm^2^, respectively. The nylon membrane manufactured through electrospinning is used to manufacture a negative electrode substrate; flexible HSC could be manufactured with high flexibility. Flexible HSCs have stable electrical output at various bending angles. Electrochemical analysis confirmed that the developed FeV-LDHs hierarchical structures deposited on sandpaper, by improving the diffusion rate of electrolyte ions into the electrode, possess excellent electrochemical supercapacitor performance.

## 2. Materials and Methods

### 2.1. Materials

Iron chloride hexahydrate (FeCl_3_·6H_2_O, 97%), vanadium chloride (VCl_3,_ 97%), nylon 6/6 pellets (Nylon), and N, N-dimethylformamide (DMF, 99.8%) were purchased from Sigma-Aldrich. The commercial sandpaper (SP) substrates were manufactured by 3M and purchased online. Activated carbon (AC), and super P carbon were procured from MTI Korea. Formic acid was obtained from Daejung-Chemicals.

### 2.2. Deposition of Thin Ni Layer on SP and Membrane Substrate

A conductive Ni layer was sputtered onto an SP and nylon membrane (NMB) substrate by a magnetron RF sputter at 150 W for 45 min in Ar gas at 30 sccm.

### 2.3. Preparation of FeV LDHs on Conductive SP Substrate

The growth solution was prepared by mixing well-dissolved solutions of FeCl_3_·6H_2_O (as an Fe precursor) and VCl_3_(as V precursor) in 30 mL of D.I. water. The ratios of each metal precursor and the concentrations of the solutions were carefully considered in the resultant synthesis process. Solutions were mixed for 1 h at room temperature. The deposition of metal hydroxides on the SP substrate was carried out by simple electrochemical deposition (ECD). Following the ECD, the SP substrate coated with electroactive material was utilized as it is as the working electrode, and platinum (Pt) wire and Ag/AgCl were employed as the counter and reference electrodes, respectively. To coat the required material uniformly on the SP substrate, a chronoamperometry voltage of −1.0 V was supplied for 100 s. Further, the electrodes were allowed to dry for 5 h at 80 °C.

### 2.4. Preparation of AC-Based Negative Electrode (AC//SP)

The AC layer was deposited on the SP substrate using the appropriate weight ratio (80:10:10) of activated carbon (AC), polyvinylidene fluoride (PVdF), and super P carbon. To increase the surface conductivity, a small amount of super P carbon was added to the mixture. PVdF was used as a binder. Slurry-type mixtures were coated onto the surface of the SP substrate and dried for 4 h at 80 °C.

### 2.5. Preparation of AC-Based Negative Electrode (AC//NMB)

The nylon pellet was dissolved in a formic acid solvent by magnetic stirring overnight at 50 °C. Furthermore, it was filled into a syringe terminated by a stainless-steel needle. The needle was held at 10 kV using an electrospinning machine (Electrospinning system, MTDI) at a distance of 10 cm from the grounded collector drum. The nylon solution was fed at a speed of 1.5 mL/h and the drum collector was spun at 500 rpm for 1 h. After electrospinning, a drying process is performed in an oven at 80 °C for 2 h. After drying, a conductive Ni layer was sputtered onto the NMB substrate, and an AC layer was deposited on the NMB substrate using the appropriate weight ratio (80:10:10) of AC, PVdF, and super P carbon.

### 2.6. Physical Characterization

The surface morphologies of the prepared electrodes were analyzed using field emission scanning electron microscopy (FE-SEM, Carl Zeiss, Oberkochen, Germany). The elemental mapping analysis was obtained by energy dispersive X-ray spectroscopy (EDX). The crystallinity of the electrodes was studied by X-ray diffractometer (XRD, D8 Advance, Bruker, Billerica, MA, USA). The chemical stoichiometry and electronic states of the electrodes were studied by an X-ray photoemission spectroscopy (XPS, Thermo Electron Multilab 2000, Thermo Fisher Scientific Inc., Waltham, MA, USA) with microfocused monochromated Al Kα X-rays.

### 2.7. Electrochemical Characterization

The electrochemical performance of all the electrodes and the supercapacitor device was tested and evaluated in a 1M aqueous KOH electrolyte solution under ambient conditions using an Iviumstat electrochemical instrument (IVIUM Technologies). Cyclic voltammetry (CV), galvanostatic charge-discharge (GCD), and electrochemical impedance spectroscopy (EIS) were recorded to examine the electrochemical performance of electrodes. The as-prepared free-standing electrodes were directly tested as the working electrodes. Platinum wire and Ag/AgCl were employed as the counter and reference electrodes, respectively. Furthermore, the values of areal capacity (*Q_ac_*) and areal capacitance (*C_a_*) were carefully calculated by the following Formulas (1) and (2) [9].

(1) $$Q_{ac}(\frac{Ah}{a})=\frac{I\times \Delta t}{a}$$

(2) $$C_{a}=\frac{I\times \Delta t}{a\times \Delta V}$$

where *I* is the applied discharge current (*A*), ∆*t* is the discharge time (s), *a* is the area of electroactive material (cm^2^), and ∆*V* is the potential window (*V*), *C_a_* is the areal capacitance (F/cm^2^), and *Q_ac_* is the areal capacity (Ah/cm^2^).

### 2.8. Fabrication of Pouch-Type HSC Device

The hybrid supercapacitor (HSC) device, consisting of a positive electrode, a negative electrode, a separator, and electrolyte, was fully sealed for two electrode configurations. The suggested FeV LDH coated on SP substrate was used as a positive electrode (+ve electrode), and activated carbon (AC) coated SP substrate and NMB substrate were used as a negative electrode (−ve electrode) for the assembly of HSC devices. These two electrodes were separated by filter paper and immersed in 1 M KOH electrolyte. Consequently, the areal capacitance (*C_a_*), areal energy density (*E_ad_*), and power density (*P_d_*) of the fabricated HSC device were estimated using the following Formulas (3) and (4) [9].

(3) $$E_{ad}=\frac{C_{a}\times \Delta V^{2}}{2}$$

(4) $$P_{d}=\frac{E_{ad}}{\Delta t}$$

where *E_ad_* is the areal energy density (Wh/cm^2^) and *P_d_* is the areal power density (W/cm^2^).

## 3. Results and Discussion

**Figure 1:**
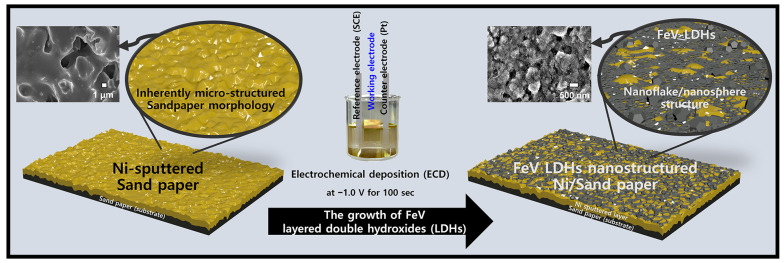
Schematic illustration of synthetic decoration of FeV layered double hydroxides (LDHs) on Ni-sputtered sandpaper (SP) substrate via facile electrochemical deposition (ECD) technique.

Schematic images of the synthetic process of the suggested Fe-V composite-based electrode are shown in Figure 1. For the development of a hierarchical micro-nano structure, inherent micro-morphological SP was utilized as a novel substrate material. Nano-structured Fe-V LDHs of electroactive materials were coated onto a Ni-sputtered SP substrate via a facile electrochemical deposition (ECD). The Fe-V growth solution was prepared with individual metal precursors of Fe and V; detailed conditions are provided in the experimental section. For Fe-V deposition, the ECD technique was used at −1.0 V fixed potential for 100 s. Following the deposition of FeV LDHs on the Ni-sputtered SP, the morphology was transformed into a nanoflake/nanosphere structure.

**Figure 2:**
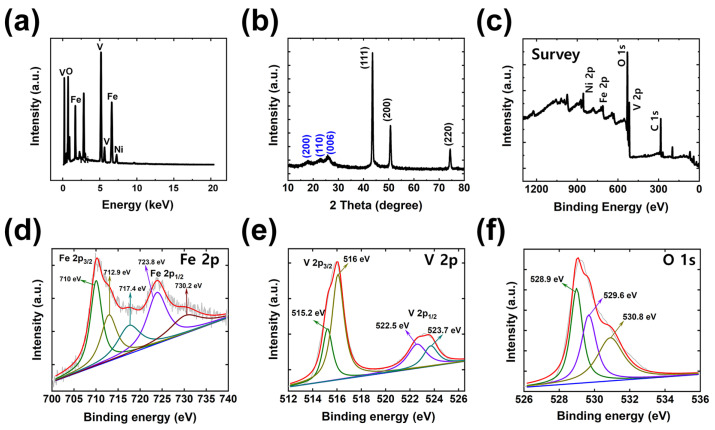
Material analysis of (**a**) EDX spectra, (**b**) XRD, and (**c**–**f**) XPS characterization of FeV LDHs: (**c**) survey, (**d**) Fe 2p, (**e**) V 2p, and (**f**) O 1s spectra.

As can be seen in Figure 2, X-ray diffraction (XRD), energy dispersive X-ray spectroscopy (EDX), and X-ray photoelectron spectroscopy (XPS) characterization were conducted to further examine the materials of the synthesized electrodes (Fe0._75_V_0.25_). The EDX spectra shown in Figure 2a indicate the existence of each element. The significant peaks of Ni, Fe, V, and O confirm the presence of these elements in the synthesized Fe_0.75_V_0.25_ electrode. Ni peaks are from the layer sputtered onto the SP substrate; the remaining peaks of Fe, V, and O are from the coated FeV LDH electroactive material.

For further analysis, an XRD study was carried out to check the phase and crystallinity, with the results shown in Figure 2b. The well-defined diffraction peaks observed at 2*θ* values of 17.2° in the (200) plane and 26.2° in the (006) plane were indexed to vanadium hydroxide. The orthorhombic crystal structure of vanadium hydroxide was consistent with the standard XRD diffraction peaks (JCPDS. 41-1426). The peak observed at a 2*θ* value of 22.5° in the (110) plane was indexed to iron hydroxide. The orthorhombic crystal structure of iron hydroxide matched available reports on iron hydroxide (JCPDS. 29-0713). Herein, XRD peaks at 2*θ* values of 44°, 51°, and 76° were well indexed to the (111), (200), and (220) planes, respectively, showing the cubic structure of the conductive Ni substrate. No other crystalline phase is detected, indicating a well-defined layered structure with high crystallinity.

XPS analysis was performed to study the chemical composition, the elemental binding state, and the surface electronic structure of the FeV LDH. Figure 2c displays the XPS survey spectra of FeV LDH, which consists of iron, vanadium, oxygen, and carbon elements. All the XPS spectra were fitted with a Shirley background. In Figure 2d, the core-level Fe 2p curves are shown, where a peak is obtained at 710 eV for Fe 2p_3/2_ and a peak at 723.8 eV for Fe 2p_1/2_. The occurrence of these peaks confirms the presence of Fe at both +2 and +3 oxidation states. Also, a peak is seen at 717.4 eV, which is a satellite peak that indicates the Fe^3+^ oxidation state [38]. Figure 2e illustrates the presence of V 2p peaks at V 2p_3/2_ and V 2p_1/2_. For V 2p_3/2_, the peaks obtained at 515.2 and 516 eV correspond to the presence of vanadium in the V^4+^ and V^5+^ oxidation states, respectively. Similarly, the V^4+^ and V^5+^ oxidation states of vanadium are observed for V 2p_1/2_ at 522.5 and 523.7 eV, respectively. The deconvoluted peaks for O 1s are illustrated in Figure 2f; also, a major peak is obtained at 528.9 eV, and two other peaks at 529.6 and 530.8 eV are attributed to the oxygen in vanadium and iron oxide [39]. In an alkaline solution of KOH electrolyte, the metal hydroxides Fe(OH)_2_ and V(OH)_3_ were easily converted to metal oxides (Fe_2_O_3_ and V_2_O_5_) by fast redox reaction. Followed by the deconvoluted O 1s spectra, the chemical reactions between metal oxide and metal hydroxide were systematically analyzed according to the following Equations (5)–(7) [1,2,3].

FeV_2_O_4_ + 4H_3_O^+^ + 4OH^−^ ⇄ Fe(OH)_2_ + 2V(OH)_3_ + 4H_2_O (5)

2Fe(OH)_2_ + 2OH^−^ ⇄ Fe_2_O_3_ + 3H_2_O + e^−^ (6)

2V(OH)_3_ + 4OH^−^ ⇄ V_2_O_5_ + 5H_2_O + e^−^ (7)

**Figure 3:**
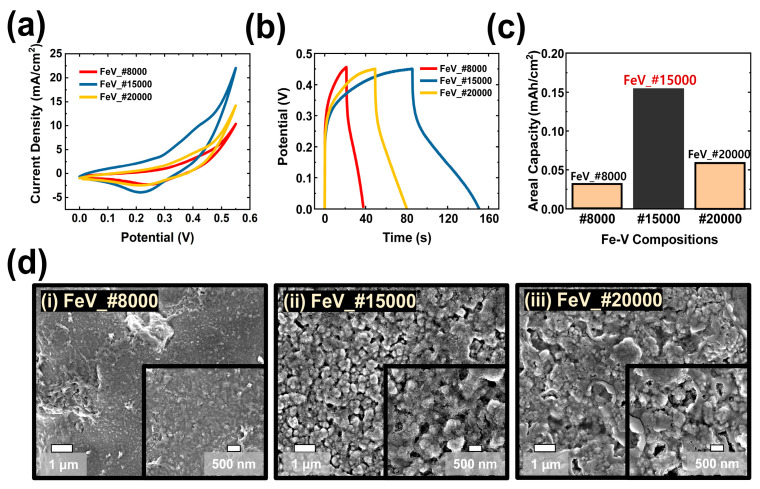
Optimization of grit number of sandpaper substrates. Comparisons of electrochemical performance of FeV-LDHs coated on sandpaper substrates of various grits (FeV_#8000, FeV_15000, and FeV_20000): (**a**) CV, (**b**) GCD, and (**c**) calculated areal capacity values for GCD results. (**d**) Comparisons of surface morphological analysis results of all materials, obtained by FE-SEM characterization.

Detailed morphological and electrochemical performance analyses of Fe-V compositions coated on various grits of SP substrate (grit numbers of #8000, #15000, and #20000) were conducted, with results shown in Figure 3. It is well known that an enlarged specific surface area of electrodes is crucial for the enhancement of electrochemical performance. Here, to create a unique hierarchically-structured electrode based on SP as the substrate, it was important to optimize the SP grit number for detailed classification of SP structure. In terms of inherent SP properties, particle size as well as the surface morphology of SP can significantly affect specific electroactive sites. Generally, various grit numbers of SP indicate the particle number per square inch. Thus, for better surface engineering, it is possible to study the effect of the optimized particle number on increasing the surface area.

To further exploit the final structures of each electrode, electrochemical measurements of Fe-V composite electrodes (coated onto different SPs with grit numbers of #8000, #15000, and #20000) were carefully performed in the three electrode configuration shown in Figure 3a–c. In Figure 3a,b, cyclic voltammetry (CV) and galvanostatic charge–discharge (GCD) plots of these electrodes (FeV_#8000, FeV_#15000, and FeV_#20000), respectively, are provided; these were obtained using 1.0 M KOH aqueous solution as electrolyte. The FeV_#15000 electrode exhibited the highest electrochemical potential among all the electrodes. The much higher capacity of the optimized FeV_#15000 was successfully demonstrated by the larger integral area of the CV graph and longer charge-discharge time of the GCD graph. To allow a systematic comparison, Figure 3c shows calculated values of areal capacity for the three electrodes. Areal capacity values of FeV_#8000, FeV_#15000, and FeV_#20000 were 0.031, 0.156, and 0.056 mAh/cm^2^, respectively. Finally, the abovementioned FeV_#15000 electrode was considered to have the optimized composition, showing the highest areal capacity due to its superior electrochemical performance.

Aside from electrochemical measurements, morphological studies of these synthesized electrodes were carefully carried out, with results shown in Figure 3d. The electrodes of FeV_#8000, FeV_#15000, and FeV_#20000 exhibited unique surface morphologies after being coated on SPs with various grit numbers. The inherently different morphologies of the SP (as substrate) lead, through the synthesis of electroactive layers (Fe-V composition), to various electroactive sites. The optimized FeV_#15000 sample exhibited double morphology structures with (i) well-designed nanosheets grown on the porous SP surface, formed by LDH (Fe(OH)_2_ and V(OH)_3_), and (ii) hierarchically-designed interconnected nanoflakes, formed by metal oxides (Fe_2_O_3_ and V_2_O_5_). XPS characterization was used to study the reaction between metal hydroxides and metal oxides; the various oxidation states of Fe and V proved the existence of these structures. Considering the chemical formulas of Fe(OH)_2_ and V(OH)_3_, the oxidation states of Fe^2+^ and V^4+^ were studied for LDHs. Also, in the chemical formulas of Fe_2_O_3_ and V_2_O_5_, the oxidation states of Fe^3+^ and V^5+^ were studied for metal oxides. All these results suggest that the unique double morphology forms due to the presence of LDH and metal oxide structures based on transition metal hydroxide/oxide active materials. Furthermore, a nanomorphology of the Fe-V hydroxides/oxides (in electroactive material) can be hierarchically coated onto the inherent micro morphology of the porous SP substrate. On the other hand, FeV_#8000 shows a surface structure with almost flat nanosheet morphology; FeV_#20000 demonstrates a porous surface with partially blocked morphology. Compared with these two electrodes (FeV_#8000 and FeV_#20000), the unique surface morphology of the optimized electrode (FeV_#15000) led to its superior charge storage performance. The appearance of this unique surface morphology for FeV_#15000 grit allows the enhancement of charge-storage performance. The large electrochemically active area of FeV_#15000 resulted in a faster electron transfer between electrolytes and electroactive materials. Accordingly, the distance of interface electrolyte ion diffusion and electron transport pathways can be easily decreased in energy storage mechanisms. Based on these results, further electrochemical measurements were acquired for the optimized SP substrate of #15000-grit.

**Figure 4:**
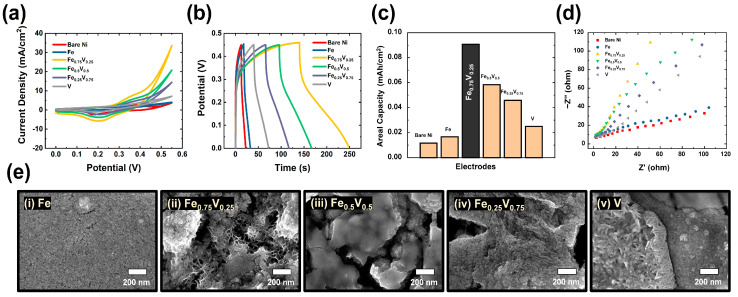
Electrochemical performance of optimized FeV composition. (**a**–**d**) Comparison of electrochemical performance of various compositions (Bare Ni, Fe, Fe_0.75_V_0.25_, Fe_0.5_V_0.5_, Fe_0.25_V_0.75_, and V) through (**a**) CV, (**b**) GCD, and (**c**) values of areal capacity calculated from GCD results; and (**d**) EIS plot. (**e**) Comparisons of obtained surface morphological analysis results for all materials, obtained by FE-SEM characterization.

To determine the proper ratio of Fe to V precursors for electroactive materials, electrochemical measurements of various Fe-V compositions coated onto an optimized #15000 grit SP substrate were conducted, with the results shown in Figure 4. The various compositions (Ni, Fe, Fe_0.75_V_0.25_, Fe_0.5_V_0.5_, Fe_0.25_V_0.75_, and V) and morphologies exhibited different levels of electrochemical performance as well as material-specific properties. The various electrochemical activities resulted from the different contributions of Fe and V in each composite material.

Figure 4a shows typical CV curves with potential ranges from 0.0 to 0.55 V at a fixed scan rate of 100 mV/s. GCD curves with a current density of 3 mA/cm^2^ are shown in Figure 4b. For comparison, the Fe_0.75_V_0.25_ electrode demonstrated the highest electrochemical performance among all compositions, with a larger CV area and longer charge-discharge times. The values of areal capacity were calculated according to the measured discharging time, applied current, and area of electroactive sites, and the highest areal capacity value of Fe_0.75_V_0.25_ was successfully obtained, as shown in Figure 4c. As the current densities increased, the areal capacity values gradually decreased according to the equation, and all the areal capacity values are provided in Table S1.

For systematic confirmation of the feasibility of transport of electrolyte ions and electrons in FeV electroactive materials, electrochemical impedance spectroscopy (EIS) of optimized Fe_0.75_V_0.25_ electrode was performed, with results shown in Figure 4d. The impedance spectra in the Nyquist plots were recorded in a frequency range of 10,000 to 0.01 Hz; materials demonstrated different behaviors in the different frequency regions. To reveal the electrochemical performance of the suggested electrode in a particular electrolyte, Nyquist plots showing an imaginary component (Z″) against the real component (Z′) were analyzed; these showed a semicircle in the high frequency region and a straight line in the low frequency region. In the high frequency ranges, the equivalent series resistance (ESR) can be obtained to determine the solution resistance (*R*_s_), the internal resistance of the active material, and the contact resistance at the active material/substrate interface; these values can be used to determine the charge transfer resistance (*R*_ct_) at the electrode/electrolyte interface. In the low frequency range, the diffusion of the electrolyte within the electrode was evaluated by examining electrolyte penetration pathways during the charge storage process. As a result, the great electrical conductivity of the optimized electrode (Fe_0.75_V_0.25_) was indicated by the small radius of its semicircle, defined using the lowest *R*_ct_, and the steeper slope of the vertical line, which is related to the characteristic of an ideal capacitor. Finally, we demonstrated the superior charge storage performance of the Fe_0.75_V_0.25_ electrode, showing that this is indeed the ideal composition. From the obtained results, the synergistic effects among different metals were proved by comparisons between mixed metal hydroxide/oxide compositions (Fe_0.75_V_0.25_, Fe_0.5_V_0.5_, and Fe_0.25_V_0.75_) and mono metal hydroxides/oxides (Fe and V). Among the mixed metal hydroxide/oxide compositions, the enhanced electrochemical performance of the optimized composited electrode made of Fe_0.75_V_0.25_ was especially proven via a detailed morphological study. The specific surface area and pore size distribution play a significant role in the electrochemical supercapacitor performance. Therefore, nitrogen adsorption–desorption isotherm studies were carried out to investigate the specific surface area and porous nature of the corresponding materials. As shown in Figure S1a, the isotherm of metal hydroxide/oxide samples with a broad hysteresis loop in the range between 0–1.0 demonstrates the mesoporous nature of the material. The Brunauer–Emmett–Teller (BET) surface area of Fe_0.75_V_0.25_ is observed to be 98.63 m^2^/g and confirmed to be the highest compared with remaining samples provided in Table S2. The enhanced surface area can offer sufficient surface sites for the faradaic redox reaction, thereby leading to enhanced electrochemical supercapacitive performance of the electrode material. Barrett–Joyner–Halenda (BJH) plot shown in Figure S1b reveals the pore size distribution of all the samples and displays an average pore size of 1.48 nm in the case of Fe_0.75_V_0.25_. This pore size can effectively support the electrolyte ions diffusion into the internal voids of the materials, contributing to their high-rate capability.

As shown in the SEM images in Figure 4e, the surface morphologies of the various compositions indicate their controllable construction; these materials promote synergistic effects of pure elements (Fe and V). The surface of the Fe-based electrode almost blocked the sandpaper pores, presenting an almost completely flat structure. Similarly, the surface of the V based electrode exhibited a simple morphology, with interconnected nanorods grown on round SP peaks. On the other hand, the electrodes made of Fe_0.5_V_0.5_ and Fe_0.25_V_0.75_ demonstrated only single morphology structures, showing smoothly-covered round peaks without significant porosity. Compared with these four samples, the optimized Fe_0.75_V_0.25_ electrode had a double morphology due to the well-designed LDH nanosheets and interconnected nanoflake structure with metal oxides. Consequently, the unique double surface morphology of Fe_0.75_V_0.25_ provides abundant active sites in the form of hierarchical nano- and micro-structures with a high surface area. By allowing a large number of electrochemical active sites, this material promotes easy access to electrolyte ions for facile charge transport in energy storage systems.

**Figure 5:**
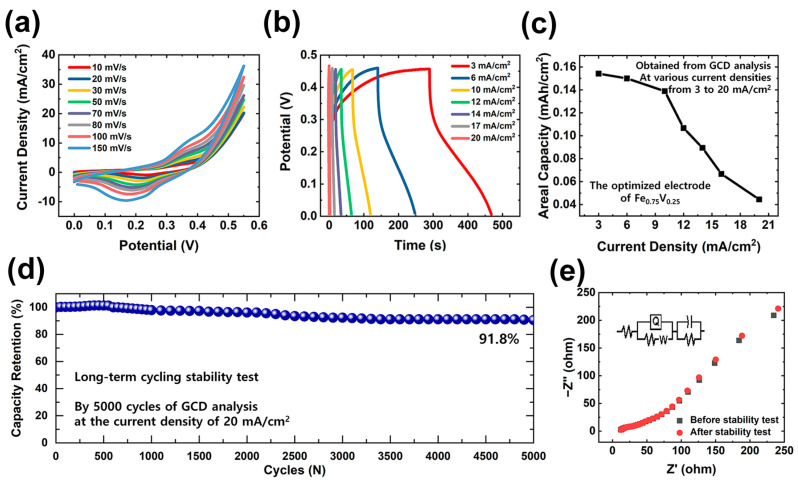
Electrochemical performance of optimized Fe-V composition. Further electrochemical properties of Fe_0.75_V_0.25__#15000 electrodes determined by (**a**) CV (from 10 to 150 mV/s) and (**b**) GCD (from 2 to 10 mA/cm^2^). Comparisons of (**c**) areal capacity values and (**d**) long-term cyclic stability test results for 5000 GCD cycles (with capacity retention of 95.1%). (**e**) The EIS curves before and after the stability test (Inset shows the electrochemical equivalent circuit).

To understand the electrochemical behavior and maximum charge storage abilities of the corresponding electroactive materials (including optimized Fe_0.75_V_0.25_), detailed electrochemical analyses were performed, with results shown in Figure 5a–d. CV curves recorded at different scan rates from 10 to 150 mV/s are shown in Figure 5a. The clearly-visible redox peaks in all the graphs indicate the electron/charge transport behavior of Fe^2+^/Fe^3+^ and V^4+^/V^5+^. The energy storage mechanism for battery-type Fe_0.75_V_0.25_ electrodes is based on these transitions between different oxidation states of Fe and V. On the surfaces of the electroactive materials, the highly hierarchical architecture provides excellent access for electrolyte ions. The integral CV area also gradually increased with the increase in the scan rate. In Figure 5b, GCD analysis results are provided that show the different current densities ranging from 3 to 20 mA/cm^2^. A uniform charging/discharging tendency, with a significant plateau shape, was recorded, indicating the reversible faradaic redox process. We calculated the areal capacity values of the Fe_0.75_V_0.25_ electrode shown in Figure 5c at different current densities ranging from 3 to 20 mA/cm^2^. Based on the abovementioned GCD results, the values of areal capacity were found to gradually decrease with the increase in the applied current density. Finally, the excellent rate performance of the suggested electrode was successfully proven via long-term stability test shown in Figure 5d. Even after 5000 GCD cycles, the electrode retained 91.8% of its initial areal capacity, indicating the good cycling stability of Fe_0.75_V_0.25_. This excellent cycling performance is a critical aspect determining electrode applicability for practical energy storage devices. To further verify the importance of electron transport in FeV LDH electrode, SEM images of the pristine electrode (Figure S2a), and after 5000 cycles (Figure S2b) are compared. As can be seen, after cycling with different charge-discharge cycles at 20 mA/cm^2^, the skeleton of FeV LDH electrode can remain intact, showing outstanding cycle life span. The EIS curve of Fe_0.75_V_0.25_ electrode before and after cyclic stability study are plotted in Figure 5e. The *R*_s_ values are 11.4 and 11.8 Ω, equivalent to the electrode before and after cyclic stability test, respectively. While the *R*_ct_ values are 10.5 and 10.3 Ω, equivalent to the electrode before and after cyclic stability test, respectively (inset of Figure 5e shows the equivalent circuit). With the slight changes in *R*_s_ and *R*_ct_, the electrode exhibits a good conductivity even after 5000 charging and discharging cycles.

**Figure 6:**
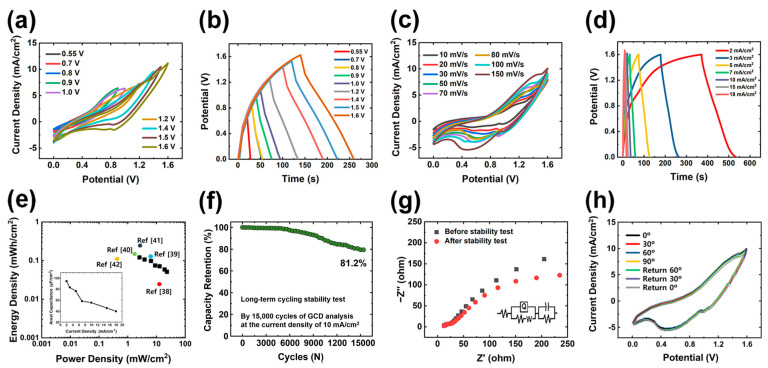
Electrochemical performance of assembled hybrid supercapacitor (HSC). Electrochemical performance under potential windows from 0.55 to 1.6 V; (**a**) CV curves (at 150 mV/s) and (**b**) GCD curves (at 3 mA/cm^2^); detailed measurement under the potential windows of 0–1.6 V in (**c**) CV graphs (from 10 to 150 mV/s) and (**d**) GCD graphs (from 2 to 18 mA/cm^2^). (**e**) Ragone plots of energy and power density of HSC device with comparison to previous works (Refs. [38,39,40,41,42]). (inset) Calculated areal capacitance values obtained by GCD measurements. (**f**) Long-term cyclic stability test for 15,000 GCD cycles (with high capacitance retention of 81.2%) and (**g**) EIS Nyquist plots (obtained from 10,000 to 0.01 Hz) before and after cyclic test. (**h**) CV plots of flexible HSC at different bending states.

Considering the superior performance of the Fe_0.75_V_0.25_ battery-type electrode, a hybrid supercapacitor (HSC), shown in Figure 6, was fabricated and studied. For the fabrication of HSC devices, the as prepared Fe_0.75_V_0.25_ and activated carbon (AC)-coated SP substrate (AC//SP) acted as battery-type positive electrodes and electric double layer capacitance (EDLC)-type negative electrodes, respectively. The preparation and electrochemical analysis of the AC//SP electrode are specified in Figure S3 (Supporting Information). To optimize the potential window of the HSC device, CV curves of the HSC device were plotted at various applied potentials varying from 0–0.6 V, 0–0.8 V, 0–1.0 V, 0–1.2 V, 0–1.4 V, and 0–1.6 V at the sweep rate of 100 mV/s, as revealed in Figure 6a. The HSC device showed a stable CV plot without any deviations up to 1.6 V, shown in Figure 6a. Similarly, the GCD analysis of the HSC device at various applied potentials ranging from 0–0.6 V, 0–0.8 V, 0–1.0 V, 0–1.2 V, 0–1.4 V, and 0–1.6 V at the current density of 3 mA/cm^2^ is plotted in Figure 6b. Further, CV and GCD results obtained at the higher applied potential window of 0–1.6 V show stable results without any deviations. Therefore, the optimum working potential window of the HSC device was fixed to be 0–1.6 V.

The CV plots of HSC device obtained at different scan rates varying from 10 to 150 mV/s within the potential window of 0–1.6 V, shown in the Figure 6c. The shape of the CV plots of the HSC represents the existence of EDLC-type and battery-type electrodes without clear redox peaks, resembling the well-balanced of both charge storage mechanisms. Figure 6d illustrates the GCD curves, which were obtained by fixing the potential range (0–1.6 V) and varying the current density from 2 to 18 mA/cm^2^. On the basis of these results, the areal capacitance values were calculated and are provided in inset Figure 6e. The values of 94.97, 83.76, 75.95, 58.27, 45.72, and 39.25 μF/cm^2^ of areal capacitance were achieved at 2, 3, 5, 7, 10, 15, and 18 mA/cm^2^, respectively. The crucial parameters of HSCs are energy density and power density, which are estimated using Equations (3) and (4), respectively. The resultant values of these parameters are plotted in the Ragone diagram shown in Figure 6e. The HSC device delivered a higher energy density of 0.123 mWh/cm^2^ and a maximum power density of 23.8 mW/cm^2^. As shown in Figure 6e, the energy and power density values of the current HSC device are higher than the values of recently reported core-shell heterostructure-based supercapacitors and are provided in Table S3 [38,39,40,41,42]. Making the use of HSCs in any practical application, cycling stability is most important. Therefore, the cycling stability study was carried out for up to 15,000 continuous GCD cycles at the current density of 10 mA/cm^2^. The HSC device delivers good stability with 81.2% capacity retention after 15,000 continuous GCD cycles, as shown in Figure 6f. The *R*_s_ values are 10.7 and 9.3 Ω, equivalent to the device before and after the cyclic stability test, respectively. While, the *R*_ct_ values are 5.2 and 4.7 Ω, equivalent to the device before and after the cyclic stability test, respectively. With the minor changes in *R*_s_ and *R*_ct_, the device revealed good conductivity even after 15,000 continuous charging and discharging cycles (Figure 6g). Such remarkable results unambiguously support the suitability of the as constructed HSC device as an exceptional aspirant for high-energy and power density applications in the energy storage sector. Figure 6h displays the CV plots of the flexible HSC under various bending angles at 150 mV/s. Regardless of the bending degree, the flexible HSC exhibited excellent electrochemical stability, thereby indicating excellent mechanical stability. The optical image of flexible HSC according to the various bending angles is shown in Figure S4.

## 4. Conclusions

In summary, FeV-LDH nano-flakes were successfully decorated onto a micro-structured conductive SP substrate. The effective decoration of FeV-LDHs, by contributing as an electroactive material, on the SP substrate allowed the synthesized electrode to facilitate the effective diffusion of electrolyte ions. With the merits of high surface area and unique structural design, the as prepared, optimized Fe_0.75_V_0.25_ LDHs demonstrated their superior electrochemical performance. Furthermore, an asymmetric capacitor composed of Fe_0.75_V_0.25_ LDHs as a faradaic electrode and activated carbon (AC) as a capacitive electrode achieved high levels of energy and power densities. Furthermore, HSC manufactured with AC coated electrospun nylon membrane as electrode showed high flexibility. Ultimately, this work shows the great potential of Fe_0.75_V_0.25_ LDHs for application to the energy storage sector with enhanced electrochemical performance.

## Data Availability

The data presented in this study are available on request from the corresponding author.

## Supplementary Materials

The following supporting information can be downloaded at: https://www.mdpi.com/article/10.3390/polym15051136/s1, Table S1: Areal capacity values of synthesized electrodes; Table S2: Surface area and pore size of synthesized samples; Table S3: Comparison of the obtained HSC device energy storage properties with the previously reported HSC/asymmetric device performances; Figure S1: BET and BJH study of all the electroactive materials; Figure S2: SEM images of FeV LDH electrode before and after cycling test; Figure S3: Preparation and electrochemical performance of AC//SP negative electrode; Figure S4: Optical image of the HSC at flat and bending states and the definition of bending angle.
