# Supplementary material for: FeV LDH Coated on Sandpaper as an Electrode Material for High-Performance Flexible Energy Storage Devices
Source: Polymers (Basel). 2023 Feb 24;15(5):1136. doi: 10.3390/polym15051136 (PMC10007251; doi:10.3390/polym15051136)
Supplement: Supplementary file 1 [file polymers-15-01136-s001.zip › polymers-2135566-supplementary.pdf]

Table of contents

S1. Areal capacity values of synthesized electrodes.
S2. BET and BJH study of all the electroactive materials.
S3. Surface area and pore size of synthesized samples.
S4. SEM analysis of FeV-LDH before and after cycling stability study.
S5. Preparation and electrochemical performance of AC//SP negative electrode.
S6. Comparison of the obtained HSC device energy storage properties with the previously reported HSC/asymmetric device performances.
S7. Optical images of HSC device at different bending positions.

S1. Areal capacity values of synthesized electrodes

Table S1. Areal capacity values of synthesized electrodes.

Sample	Areal capacity (mAh/cm²)
Bare Ni	0.011
Fe	0.016
$Fe_{0.75}V_{0.25}$	0.091
$Fe_{0.5}V_{0.5}$	0.058
$Fe_{0.25}V_{0.75}$	0.045
V	0.025

S2. BET and BJH study of all the electroactive materials.

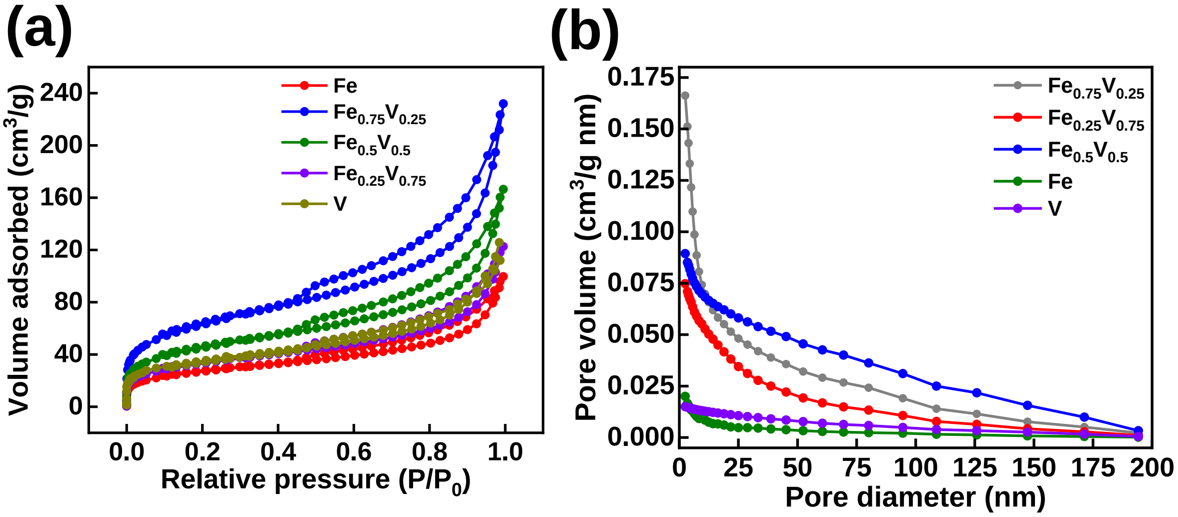

Figure S1. (a) Brunauer-Emmett-Teller (BET) nitrogen adsorption-desorption isotherm and (b) Barrett-Joyner-Halenda (BJH) pore size distribution curve of all the electroactive materials (i.e., Fe, $Fe_{0.75}V_{0.25}$, $Fe_{0.5}V_{0.5}$, and $Fe_{0.25}V_{0.75}$, and V).

S3. Surface area and pore size of synthesized samples.

Table S2. Surface area and pore size of synthesized samples.

Sample	Surface area (m²/g)	Pore size (nm)
Fe	21.55	12.07
$Fe_{0.75}V_{0.25}$	98.63	1.48
$Fe_{0.5}V_{0.5}$	63.21	5.53
$Fe_{0.25}V_{0.75}$	44.58	6.52
V	36.21	9.78

S4. SEM analysis of FeV-LDH before and after cycling stability study.

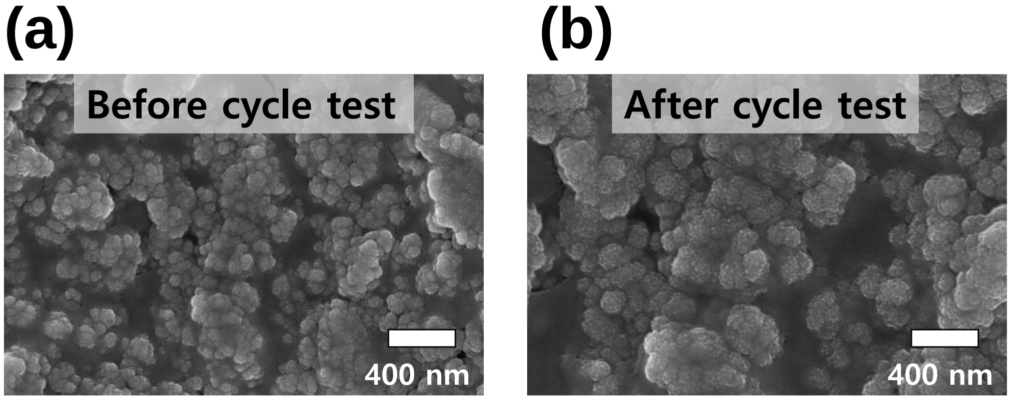

Figure S2. SEM images of FeV LDH electrode (a) before GCD cycle stability study, (b) after 5,000 GCD cycles at 20 mA/cm².

S5. Preparation and electrochemical performance of AC//SP negative electrode.

Deposition of activated carbon on sand paper (AC//SP) was carried out using a simple slurry coating method. The active material (AC), super P carbon, and PVDF binder were reserved in a weight ratio of 80:10:10 and mixed thoroughly using an agate mortar with the help of few drops of N-methyl 2-Pyrrolidone (NMP) solvent till the thick uniform slurry formation. Then the slurry was distributed on sand paper substrate (1x1 cm²) followed by vacuum drying at 80 ℃ for 4 h. Electrochemical properties of AC//SP negative electrode were explored with the aid of cyclic voltammetry (CV), galvanostatic charge/discharge (GCD) in 1 M KOH electrolyte using an Iviumstat electrochemical workstation (IVIUM Technologies). CV plots were recorded at various sweep rates ranging from 10 to 50 mV/s within a potential window of -1 to 0 V. As explained from Figure S1a, the CV plots are in quasi-rectangle shapes, signifying the capacitive behavior of the electrode. Similar behavior is detected in GCD plots of the electrode recorded at different current densities varying from 3 mA/cm² to 10 mA/cm² (Figure S1b) with triangular shaped curves.

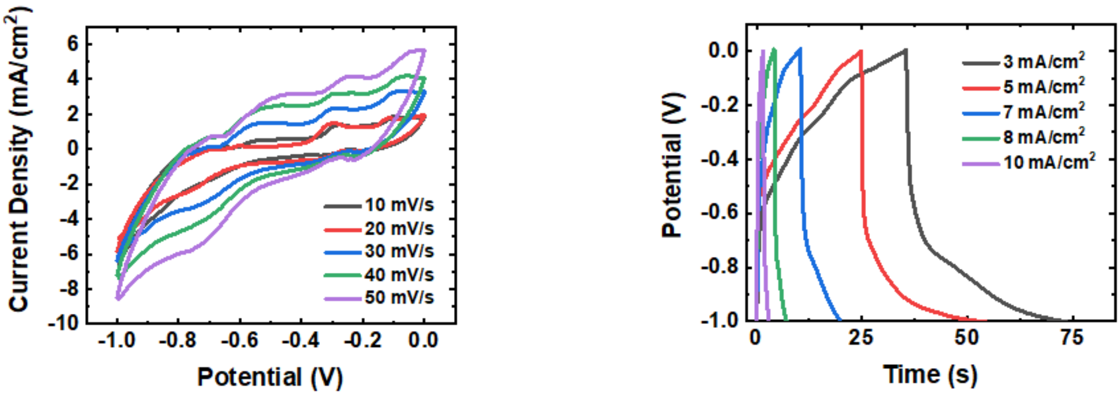

Figure S3. (a) CV curves of AC//SP electrode at different sweep rates varying from 10 to 50 mV/s, and (b) GCD curves of AC//SP electrode at various current densities ranging from 3 to 10 mA/cm².

S6. Comparison of the obtained HSC device energy storage properties with the previously reported HSC/asymmetric device performances.

Table S3. Comparison of the obtained HSC device energy storage properties with the previously reported HSC/asymmetric device performances.

References	Substrate	Electrolyte	Active material	Energy density	Power density
Ref [38]	Ni foam	2M KOH	MnFe	44.9 μW h/cm^2	16 mW/cm^2
Ref [39]	Ni foam	2M KOH	ZnCo	0.22 mW/cm^2	9 mW/cm^2
Ref [40]	Ni foam	2M KOH	NiAl	0.22 mWh/cm^2	4.5 mW/cm^2
Ref [41]	Ni foam	2M KOH	Ag	0.569 mWh/cm^2	3.82 mW/cm^2
Ref [42]	Ni foam	3M KOH	Co_2Al	0.102 mWh/cm^2	0.75 mW/cm^2
Our study	Sand paper	1M KOH	$Fe_{0.75}V_{0.25}$	0.123 mWh/cm^2	23.8 mW/cm^2

S7. Optical images of HSC device at different bending positions.

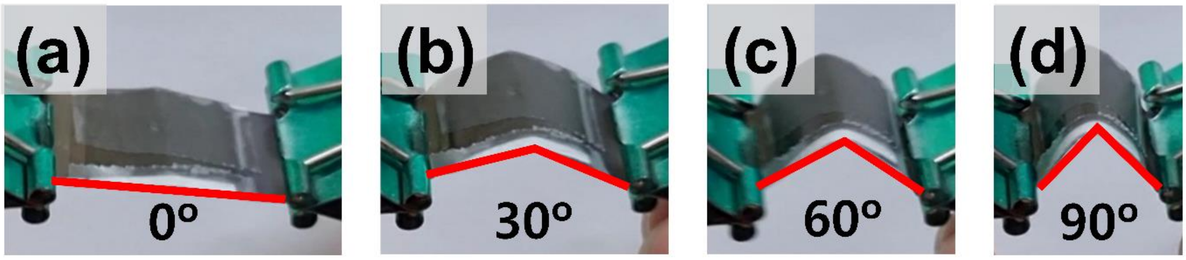

Figure S4. Optical image of the HSC at flat and bending states and the definition of bending angle. (a) flat state, bending angle (b) 30º, (c) 60º, and (d) 90º.
